# Direct and indirect effects of enablers on HIV testing, initiation and retention in antiretroviral treatment and AIDS related mortality

**DOI:** 10.1371/journal.pone.0172569

**Published:** 2017-02-22

**Authors:** Ali Safarnejad, Jose-Antonio Izazola-Licea

**Affiliations:** 1 Evaluation team, Evaluation and Economics Division, Strategic Information and Evaluation Department, United Nations Programme on AIDS (UNAIDS), Geneva, Switzerland; 2 Chief of the Evaluation and Economics Division, Strategic Information and Evaluation Department, Programme Branch, United Nations Programme on AIDS (UNAIDS), Geneva, Switzerland; University of British Columbia, CANADA

## Abstract

**Background:**

An enabling environment is believed to have significant and critical effects on HIV and AIDS program implementation and desired outcomes. This paper estimates the paths, directionality, and direct and indirect associations between critical enablers with antiretroviral treatment (ART) coverage and to AIDS-related mortality.

**Methods:**

Frameworks that consider the role of enablers in HIV and AIDS programs were systematically reviewed to develop a conceptual model of interaction. Measurements for constructs of the model were pooled from the latest publicly available data. A hypothetical model, including latent/unobserved factors and interaction of enablers, program activities and outcomes, was analyzed cross-sectionally with structural equation modeling. Coefficients of the model were used to estimate the indirect associations of enablers to treatment coverage and the subsequent associated impact on AIDS related mortality.

**Findings:**

The model’s fit was adequate (RMSEA = 0·084, 90% CI [0·062, 0·104]) and the indirect effects of enablers on outcomes were measured. Enablers having significant associations with increased ART coverage were social/financial protection, governance, anti-discrimination, gender equality, domestic AIDS spending, testing service delivery, and logistics.

**Interpretation:**

Critical enablers are significantly correlated to outcomes like ART coverage and AIDS related mortality. Even while this model does not allow inference on causality, it provides directionality and magnitude of the significant associations.

## Introduction

Substantial scale-up in resources and coverage of services have been observed in low- and middle-income countries in recent years to total an estimated US$19·14 Billion invested including services for 13·6 million people receiving antiretroviral treatment as of June 2014 [1]. More ambitious targets are being proposed by the United Nations AIDS Program (UNAIDS) to achieve by 2030 a 90% decline in number of new HIV infections and AIDS-related deaths compared to 2010, alongside “zero” discrimination [1].

While targets are set and needed investments estimated, debate continues over the individual and meso-level factors that affect the performance of HIV and AIDS programs. Reports suggest these social, political, economic and developmental enablers (heretofore referred to as “enablers”) have a significant effect on program activities in achieving the objectives of the AIDS response [2–4]. Some researchers view the socio-cultural, political and economic factors as drivers of the epidemic and posit the importance of these structural determinants in the success of HIV and AIDS programs [5, 6]. Others have studied the proximate effect of social, political and structural enablers on increased rate of testing, treatment coverage, adherence, or retention [4, 7–11]. There is, however, a gap in research on the combined interaction effect of enablers on the AIDS response, and measurement of their total (direct and indirect) effects on program objectives.

In the present study, we specify a model for the effect of enablers on program activities and outcomes, guided by a combination of theory and exploratory analysis. We test the hypothesized model’s fit to empirical data, and examine its ability to predict the effect of enablers on program activities and outcomes. We also examine the pathway of effect and the size of the total effect of enablers on outcomes and program activities.

To our knowledge, this is the first study to examine the interaction effects of multiple enablers on program activities and outcomes in the AIDS response, using structural equation modeling. It articulates the enablers relevant to an effective AIDS response, and weighs their impact on program activities and outcomes.

## Methods

A systematic review identified frameworks of the program response that consider the role of enablers [2, 12–14]. The frameworks were synthesized to formulate a conceptual model of relationship between enablers, program activities, and outcomes (not shown). Table 1 presents all constructs and variables with available data considered in the conceptual model, the conceptual framework which reference the construct, decisions regarding its inclusion into the final hypothesized model, and the year or range of years for the indicators. Efforts were made to include the latest available data, allowing for a time delay between the critical enablers and changes in outcomes of treatment coverage and mortality.

**Table 1:** Constructs considered in conceptual model, corresponding variables of their measurement, data sources, and decisions with respect to inclusion of variable in the model.

Construct	Related Variables	Data Source
ART Retention	**Twelve month retention on antiretroviral therapy**	UNAIDS: http://aidsinfo.unaids.org
HIV Prevalence	**Estimated number of people living with HIV per total population**	Global report: UNAIDS report on the global AIDS epidemic 2013. Annex: Pp A3-A9. UNAIDS: Geneva, 2013
Human Rights and Anti-Discrimination+	**Status of International Covenant on Civil & Political Rights (ICCPR); Status of the International. Covenant on Economic, Social & Cultural Rights (ICESCR); Status of International Convention on Elimination of All Forms of Racial Discrimination (ICERD); Status of International Convention on Elimination of All Forms of Discrimination against Women (CEDAW); Convention on the Rights of the Child (CRC);** Convention on the Rights of Persons with Disabilities (CRPD)^a^; Convention against Torture and Other Cruel Inhuman or Degrading Treatment or Punishment (CAT)^a^; Convention for the Protection of All Persons from Enforced Disappearance (CED)^a^; International Convention on the Protection of the Rights of All Migrant Workers and Members of Their Families (CMW)^a^; **Reporting on International Covenant on Rights & Discrimination;**	OHCHR: http://tbinternet.ohchr.org/
Financing*	**Domestic Public Expenditure on AIDS from Public Sources** (UNAIDS, 2011) / **Total Health Expenditure** (WB, 2011)	UNAIDS: http://aidsinfo.unaids.org
Gender+	**Gender Inequality Index;** Prevalence of recent intimate partner violence ^c^; Gender Scorecard: **Disaggregated Data, Qualitative Assessments,** National Response Gender Review ^a^, GBV/HIV Data ^a^, **Data on National Responses for Women’s Programmes**, Funding for men/boys programmes ^a^, **SRH-HIV integration, Health Sector GBV Policy, HIV plans/budgets in women ministries,** Female condoms ^a^, **HIV**+ **women participation in response review, Affected women participation in CEDAW monitoring, Social protection for** +**women,** Response budget for women organizations ^a^;	UNDP: http://hdr.undp.org/…/gender-inequality-index-gii UNAIDS: http://aidsinfo.unaids.org
Governance*	Voice and Accountability ^e^; Political Stability and Absence of Violence ^e^; **Government Effectiveness; Regulatory Quality; Rule of Law; Control of Corruption;**	The World Bank: http://www.govindicators.org
Health Workforce*	Number of nursing personnel and physicians per 1,000 people ^e^	WHO: http://apps.who.int/…/node.main.HWF
HIV Testing & Counseling	**Number who received HIV Testing and Counseling (>15 years) per 1000 people**	WHO: http://apps.who.int/…/node.main.625
Human Development#+	Mortality rate, infant (per 1,000 live births) ^b^^,^ ^e^; Literacy rate, adult total (% of people ages 15 and above) ^b^^,^ ^e^; Population with at least secondary education ^c^; Employment to Population Ratio ^c^; Multidimensional Poverty Index Value ^b^^,^ ^e^; Percent of population in multidimensional poverty ^b^^,^ ^e^; **Human Development Index;**	UNDP: https://data.undp.org
Legal Environment+	**Laws that specifically criminalize HIV transmission or exposure; Laws deeming sex work («prostitution») to be illegal; Laws that impose compulsory treatment for people who use drugs and/or provide for death penalty for drug offences; Laws, regulations or policies that present obstacles to access to prevention, treatment, care and support for vulnerable subpopulations;** Laws that criminalize same-sex sexual activities between consenting adults ^a^; HIV-specific restrictions on entry, stay or residence ^a^; Laws and regulations that protect people living with HIV against discrimination ^a^; Non-discrimination laws or regulations that specify protections for vulnerable subpopulations ^a^;	UNAIDS: http://www.unaids.org/…HRPoster.pdf
ART	**Antiretroviral therapy coverage based on WHO 2010 guidelines**	WHO: http://www.who.int/gho/hiv/…/ART
Logistics+	The efficiency of customs and border management clearance (“Customs”) ^e^; **The quality of trade and transport infrastructure (“Infrastructure”); The ease of arranging competitively priced shipments (“International Shipments”); The competence and quality of logistics services—trucking, forwarding, and customs brokerage (“Logistics competence”); The ability to track and trace consignments (“Tracking and tracing”); The frequency with which shipments reach consignees within scheduled or expected delivery times (“Timeliness”);**	The World Bank: http://lpi.worldbank.org/international
Mortality Rate	**AIDS Related Deaths (all ages) / Number of people living with HIV**	UNAIDS: http://aidsinfo.unaids.org
Testing Service Delivery*+	**Number of facilities in the country providing HIV testing and counseling, per 100,000 population**	WHO: http://apps.who.int/…/node.main.625
Social/Financial Protection*+	**Out of pocket expenditure on health (in current US$ per capita) / GNI per Capita (Atlas Method)**	WHO: http://apps.who.int/nha/database/Home/Index
Stigma / Homophobia+	**Comparative Homophobia Index;** Stigma Index ^c^;	ILGA: http://ilga.org Stigma Index: http://www.stigmaindex.org
TB+HIV Co-Treatment	Co-Management of TB and HIV Treatment ^b^	UNAIDS: http://aidsinfo.unaids.org

Notes.

The variables actually included in the final model are highlighted in bold format.

* Construct from Health Systems Framework

^+^ Construct from Investment Framework

^#^ Construct from Proximate-Determinants Framework

^a.^ Variable failed to group during cluster analysis

^b.^ Variable not used in model due to low significance of effect

^c.^ Variable not considered due to small sample size

^d.^ Variable rejected in factor analysis

^e.^ Inclusion of variable reduces the overall fit of the model

A measurement model was specified for the latent variables of logistics and governance, and estimated by confirmatory factor analysis [15, 16]. Empirical, cross-sectional, national level data was collected for the constructs in the model. The conceptual model was revised based on availability of data for constructs and comparability across countries, and combined with the measurement model of latent constructs to form a Structural Equation Model (SEM). Constructing variables that are proportions of absolute measures and standard denominators creates weighted constructs, which allow for comparability across countries. The latest available data was used, allowing for time delay between the critical enablers and hypothesized changes in the outcomes. The measures of constructs of the SEM are presented in Table 2.

**Table 2:** Constructs of the structural model, corresponding variables and year of their measurement.

Construct	Method of Measurement	Year(s)
AIDS Mortality	Number of AIDS Related Deaths per Number of people living with HIV (all ages)	2012
Anti-Discrimination Conventions	Ordinal measure variables constructed from five indicators: ICCPR, ICESCR, ICERD, CEDAW, CRC (see Table 1 for explanation of acronyms). Values assigned are 2: State Party, 1: Signatory (not yet Party), 0: No action. Mean of un-weighted linear sum of variables is taken as a continuous measure on Anti-discrimination Conventions.	2013
ART Coverage	Percentage of eligible adults and children currently receiving Antiretroviral therapy based on WHO 2010 guidelines	2011
Commitment to Anti-Discrimination Conventions	Total number of reports submitted on ICCPR, ICESCR, ICERD, CEDAW, and CRC. All measures are from year of ratification of convention to present time.	2014
Domestic AIDS Spending	Domestic Public Expenditure on AIDS from Public Sources per USD 1,000 of Total Health Expenditure	2011
Gender Inequality Index	Gender Inequality Index	2012
Gender Visibility Scorecard	Ordinal measure variables constructed from nine indicators of the Gender Scorecard: 1) Disaggregated Data; 2) Qualitative Assessments; 3) Data on National Responses for Women’s Programmes; 4) SRH-HIV integration; 5) Health Sector GBV Policy; 6) HIV plans/budgets in women ministries; 7) HIV+ women participation in response review; 8) Affected women participation in CEDAW monitoring; 9) Social protection for +women. Values assigned are 2: Present at National Level, 1: Available on Project Basis, 0: Not Available. Mean of un-weighted linear sum of variables is taken as a continuous measure on Gender Visibility Scorecard.	
Governance	Latent variable factored together from four indicators: 1) Regulatory Quality; 2) Control of Corruption; 3) Rule of Law; 4) Government Effectiveness;	2012
HIV Prevalence	Estimated number of people living with HIV per total population	2012
HIV Testing & Counseling	Number who received HIV Testing and Counseling (>15 years) per 1000 people	2010
Human Development Index	Human Development Index	2012
Logistics	Latent variable factored together from six indicators: 1) Customs; 2) Infrastructure; 3) International shipments; 4) Timeliness; 5) Tracking and tracing; 6) Logistics competence;	2012
Punitive Laws & Homophobia	Index constructed from five themes of the legal environment: 1) Laws that specifically criminalize HIV transmission or exposure; 2) Laws deeming sex work («prostitution») to be illegal; 3) Laws that impose compulsory treatment for people who use drugs and/or provide for death penalty for drug offences; 4) Laws, regulations or policies that present obstacles to access to prevention, treatment, care and support for vulnerable subpopulations; 5) Legal situation of lesbian, gay, bisexual and trans people («comparative homophobia index»). Each element of the index given a score of 1 or -1 based on existence of laws or policies that act as enablers or barriers respectively. A special value of -2 given to States that exercise the death penalty for drug offences. The index is the mean of un-weighted linear sum of the individual theme scores.	2010 (homo-phobia index from 2012)
Retention on ART	Percentage of adults and children with HIV known to be on treatment 12 months after initiation of antiretroviral therapy	2011–2012
Social/Financial Protection	Out of pocket expenditure on health (in current US$ per capita) per GNI per Capita (Atlas Method)	2011
Testing Service Delivery	Estimated number of facilities in the country providing HIV testing and counseling, per 100,000 population	2010

The SEM was tested using a maximum likelihood estimator [17]. The following model fit statistics were evaluated: (1) the overall chi-square test statistics for the null hypothesis that the model is consistent with the data [18], (2) the Bayesian Information Criterion (BIC) for which a negative value indicates the model is preferred over the saturated model allowing for all variables to be inter-correlated [19, 20], (3) the Comparative Fit Index (CFI) and Tucker Lewis Index (TLI) for which values greater than 0.9 are generally taken as indicative of good fit [21, 22], and (4) the Root Mean Square Error of Approximation (RMSEA) for which values less than 0.1 are indicative of good fit in small sample sizes [23, 24]. Model re-specifications were tested and the modifications were retained if they significantly improved the overall model fit as measured by fit statistics. Standardized coefficients of the final model were used to estimate the magnitude of indirect effects from a number of pathways by which enablers and program activities are associated with the treatment coverage outcomes [25]. As AIDS mortality is estimated using antiretroviral treatment (ART) coverage data in epidemiological projection packages [26], critical enablers were correlated only with ART coverage and not to AIDS related deaths. The association between ART and AIDS mortality was then estimated.

## Results

The analysis used a cross sectional dataset containing 59 observations (countries) with complete data on all relevant variables. These observations represent 13 countries in Asia and the Pacific, 9 in Latin America and the Caribbean, 7 countries in Eastern Europe and Central Asia, 25 countries in Sub-Saharan Africa, and 5 countries in the Middle East and North Africa. The countries analyzed by income level were 29 in the low-, 26 in the lower-middle- and 4 in the upper-middle-income brackets (Table 3) [27].

**Table 3:** Countries, income groups, and regions with complete data included in the analysis.

Country	Income Group	Region	HIV Prevalence (%) Adults 15–49 (2012)
Afghanistan	Low	Asia and the Pacific	<0.1 (<0.1–<0.1)
Angola	Lower–middle	Sub-Saharan Africa	2.3 (1.9–2.8)
Armenia	Lower–middle	Eastern Europe and Central Asia	0.2 (0.2–0.3)
Belarus	Lower–middle	Eastern Europe and Central Asia	0.4 (0.4–0.5)
Benin	Low	Sub-Saharan Africa	1.1 (1.0–1.3)
Bolivia	Lower–middle	Latin America and the Caribbean	0.3 (0.1–0.4)
Botswana	Upper–middle	Sub-Saharan Africa	23.0 (21.8–24.4)
Burundi	Low	Sub-Saharan Africa	1.3 (1.0–1.5)
Côte d'Ivoire	Low	Sub-Saharan Africa	0.8 (0.5–1.5)
Cambodia	Low	Asia and the Pacific	4.5 (4.1–4.9)
Cameroon	Lower–middle	Sub-Saharan Africa	2.8 (2.5–3.0)
Congo	Low	Sub-Saharan Africa	3.2 (2.8–3.8)
DR Congo	Low	Sub-Saharan Africa	0.7 (0.6–0.8)
Dominican Republic	Lower–middle	Latin America and the Caribbean	1.1 (1.0–1.2)
Ecuador	Lower–middle	Latin America and the Caribbean	0.6 (0.4–1.1)
Egypt	Lower–middle	Middle East and North Africa	<0.1 (<0.1–<0.1)
Ethiopia	Low	Sub-Saharan Africa	1.3 (1.2–1.5)
Fiji	Lower–middle	Asia and the Pacific	0.2 (0.2–0.2)
Gambia	Low	Sub-Saharan Africa	1.3 (0.9–1.7)
Georgia	Lower–middle	Eastern Europe and Central Asia	0.3 (0.2–0.4)
Ghana	Low	Sub-Saharan Africa	1.4 (1.2–1.6)
Guatemala	Lower–middle	Latin America and the Caribbean	0.7 (0.4–1.5)
Guinea-Bissau	Low	Sub-Saharan Africa	3.9 (2.9–5.3)
Guyana	Lower–middle	Latin America and the Caribbean	1.3 (0.8–2.1)
Haiti	Low	Latin America and the Caribbean	2.1 (1.9–2.3)
Honduras	Lower–middle	Latin America and the Caribbean	0.5 (0.4–0.7)
Indonesia	Lower–middle	Asia and the Pacific	0.4 (0.3–0.7)
Iran	Lower–middle	Middle East and North Africa	0.2 (0.1–0.2)
Kenya	Low	Sub-Saharan Africa	6.1 (5.9–6.3)
PDR Lao	Low	Asia and the Pacific	23.1 (21.7–24.7)
Lesotho	Lower–middle	Sub-Saharan Africa	0.9 (0.7–1.1)
Liberia	Low	Sub-Saharan Africa	10.8 (10.2–11.4)
Malawi	Low	Sub-Saharan Africa	0.4 (0.3–0.6)
Malaysia	Upper–middle	Asia and the Pacific	<0.1 (<0.1–<0.1)
Maldives	Lower–middle	Asia and the Pacific	1.2 (1.2–1.3)
Mauritius	Upper–middle	Sub-Saharan Africa	0.7 (0.6–0.9)
Moldova	Lower–middle	Eastern Europe and Central Asia	0.1 (0.1–0.2)
Morocco	Lower–middle	Middle East and North Africa	0.3 (0.2–0.4)
Nepal	Low	Asia and the Pacific	0.5 (0.4–0.6)
Niger	Low	Sub-Saharan Africa	3.1 (2.8–3.5)
Nigeria	Low	Sub-Saharan Africa	0.5 (0.4–0.7)
Papua New Guinea	Low	Asia and the Pacific	0.3 (0.2–0.6)
Paraguay	Lower–middle	Latin America and the Caribbean	0.3 (0.2–0.3)
Peru	Lower–middle	Latin America and the Caribbean	0.4 (0.2–1.3)
Philippines	Lower–middle	Asia and the Pacific	<0.1 (<0.1–<0.1)
Rwanda	Low	Sub-Saharan Africa	2.9 (2.6–3.2)
Sao Tome & Principe	Low	Sub-Saharan Africa	1.0 (0.8–1.4)
Sierra Leone	Low	Sub-Saharan Africa	1.5 (1.0–2.1)
South Africa	Upper–middle	Sub-Saharan Africa	17.9 (17.3–18.4)
Sri Lanka	Lower–middle	Asia and the Pacific	<0.1 (<0.1–<0.1)
Tajikistan	Low	Eastern Europe and Central Asia	0.3 (0.2–0.6)
Tanzania	Low	Sub-Saharan Africa	5.1 (4.6–5.7)
Thailand	Lower–middle	Asia and the Pacific	1.1 (1.0–1.2)
Togo	Low	Sub-Saharan Africa	2.9 (2.5–3.5)
Tunisia	Lower–middle	Middle East and North Africa	<0.1 (<0.1–<0.1)
Ukraine	Lower–middle	Eastern Europe and Central Asia	0.9 (0.7–1.0)
Uzbekistan	Low	Eastern Europe and Central Asia	0.2 (0.2–0.2)
Viet Nam	Low	Asia and the Pacific	0.4 (0.1–0.8)
Yemen	Low	Middle East and North Africa	0.1 (<0.1–0.3)

### Confirmatory factor analysis

**Fig 1:**
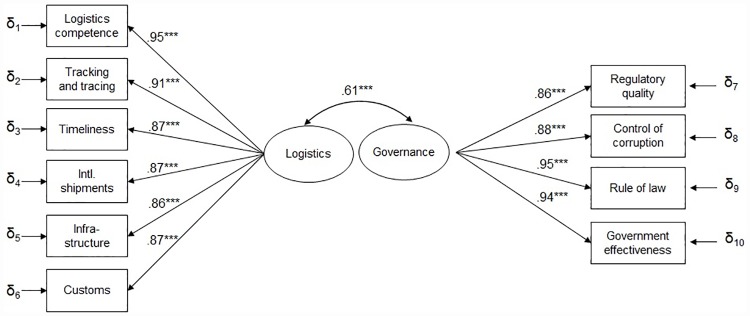
Measurement model for latent variables with standardized estimates. Diagram Elements: Boxes are manifest (observed) variables; Circles are latent (unobserved) constructs; Straight arrows point from latent constructs to measurement variables; Curved arrows are unexplained covariance among variables; δ (small delta) are residual variances of the observed variables; Values are standardized regression coefficients and number of stars signify their p-value: * p <.1; ** p <.05;*** p <.01.

Confirmatory factor analysis for the measurement model of the enabler latent constructs indicates an excellent fit of the model with the data. The chi-square test for model fit is non-significant (χ2 = 43·719, df = 34, p-value = 0·1228), the BIC is 97·032, the CFI and TLI are both 0·98, and the RMSEA is 0·07; the standardized parameter estimates for the measurement model are provided in Fig 1.

### Structural equation model

**Fig 2:**
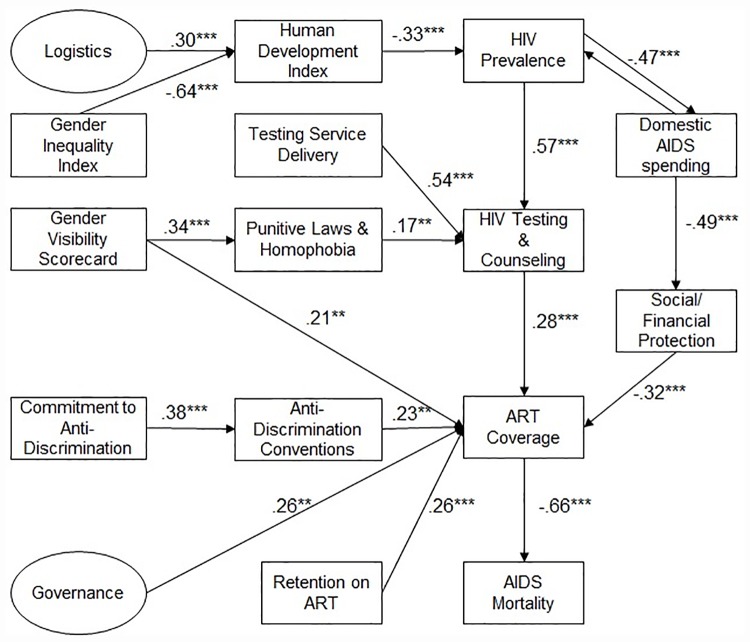
Structural equation model specifying key relationships between enablers, program activities, and outcome with standardized coefficients. Diagram Elements: Boxes are manifest (observed) variables; Circles are latent (unobserved) constructs; Arrows point from explanatory variable to dependent variable; Values are standardized regression coefficients and number of stars signify their p-value: * p <.1; ** p <.05; *** p <.01.

The hypothetical model containing all observed and latent constructs, and the relationships between the constructs, representing the hypotheses, was evaluated as a SEM. Following exhaustion of re-specifications, the final standardized model as estimated by Mplus version 7.3 [17], converged normally after 492 bootstrap draws. The final model contains 16 constructs defined by 41 variables, including two latent constructs (Fig 2). Examination of the model fit indices suggests that the structural model demonstrates an adequate fit to the data, with BIC = -183·388, CFI = 0·906, TLI = 0·894, and RMSEA = 0·084 (90% CI 0·062–0·104). The R^2^ of the model for ART coverage = 0.484, indicating that the model is explaining close to half of the variance in this key outcome. Together these statistics provide evidence for the reliability and convergent validity of the SEM. Data, model specification and model outputs are available in the supporting information S1 to S6 Files.

The model supports the hypothesis that anti-discrimination, social/financial protection, gender equality, and good governance and logistics are significant enablers of coverage of people living with HIV and AIDS on treatment. The results show that commitment to anti-discrimination had a positive indirect effect on the treatment outcome by way of conventions on anti-discrimination to which States are parties. Domestic AIDS spending with respect to total health spending positively influences treatment coverage through increased social/financial protection. The theoretical model also specifies a reciprocal interaction between domestic AIDS spending and HIV prevalence.

Governance, correlated positively with logistics, had a direct positive influence on creating an enabling environment for treatment coverage and retention. Four indicators best described governance: regulatory quality, control of corruption, rule of law, and government effectiveness. Indicators for political stability and absence of violence/terrorism and voice and accountability were dropped due to weak factoring with other governance indicators and for reducing the overall fit of the model respectively.

HIV prevalence and testing service delivery are positively associated with HIV testing and counseling in the model, while punitive laws and homophobia are negatively associated with HIV testing and counseling. Cluster analysis identified one stratum of four punitive laws and policies, with a significant explanatory power for variations in HIV testing and counseling. Cluster analysis also identified one stratum of nine variables from the gender scorecard, with significant explanatory power for variations in treatment coverage. The stratum, identified as “gender visibility scorecard” in the model, is indirectly related to HIV testing and counseling through punitive laws and homophobia.

### Standard effects

The standardized effects of each construct on the outcome variable were derived. These coefficients elucidate the direct and indirect effects of enablers and program activities on treatment coverage, which can in turn be used to estimate changes in AIDS related mortality. Table 4 lists the constructs in the model, rank sorted by the absolute value of the coefficients with ART coverage. The coefficients can be interpreted as a one standard deviation change in a critical enabler being related to one standard deviation change in ART coverage multiplied by the coefficient.

**Table 4:** Standardized effect of constructs on ART coverage derived by structural equation modeling.

Variable	Standardized Coefficient	p-value
Social/Financial Protection	-0.319	0.001
Governance	0.313	0.002
Domestic AIDS Spending	0.31	<0.001
HIV Testing & Counseling	0.274	0.009
ART Retention	0.257	0.007
Anti-discrimination Conventions	0.246	0.017
Gender Visibility Scorecard Index	0.217	0.085
Testing Service Delivery (HTC Facilities)	0.148	0.028
Human Development Index	-0.076	0.056
Commitment to Anti-Discrimination	0.072	0.123
Punitive Laws & Homophobia	0.047	0.123
Logistics	-0.023	0.100

Not all hypothesized relationships between the constructs in the model were significant. The study could not show a significant correlation between TB/HIV co-treatment, health workforce or donor disbursements per person living with HIV and program outcomes. Concerning a number of variables, there was not a sufficiently large sample size (country-wise) to warrant their inclusion without adversely affecting the overall statistical power of the entire model. For example, the intersection of data on intimate partner violence, gender-based violence, unemployment, poverty, education and data from the Stigma Index would have resulted in an inadequate ratio of observations to parameters, and therefore their hypothesized effect on the program outcomes was not collectively tested.

The association between ART coverage and AIDS related mortality was estimated, with a standardized coefficient of -0.665. This coefficient can be interpreted as one standard deviation change in treatment coverage being related to -0.665 of a standard deviation change in AIDS related deaths as a proportion of people living with HIV.

## Discussion

In view of the frameworks of the AIDS response, the effect of enablers on program activities to achieve desired outcomes is significant. This study analyzes the variables that might have a mediating effect between HIV diagnosis and treatment attrition with AIDS related deaths [28, 29]. It further elucidates the role of structural factors in creating an enabling environment to achieve maximum effectiveness of interventions by modeling the interaction of enablers, program activities, and outcomes.

Findings of the study show that gender equality, anti-discrimination, social/financial protection, governance, and logistics were significantly related to coverage and retention on antiretroviral treatment. Testing service delivery, homophobia, and punitive laws were significantly related to HIV testing and counseling. A number of other factors were found to have indirect effects on the program response. Domestic AIDS spending with respect to total health spending is positively associated with treatment coverage through lower out-of-pocket expenditures suggesting that social/financial protection may be alleviating the burden that could preclude or be an obstacle for those who test positive to seek treatment.

Gender visibility was found to be indirectly associated with increased rates of HIV testing and counseling, through its relationship with homophobia and punitive laws. This relationship suggests that consideration of gender differences and mainstreaming of gender norms is a preceding stage to reduced stigma and greater human rights for marginalized/discriminated populations. This staging of enablers resembles the structural models of development theory, such as Rostow’s Stages of Growth [30], which follow a linear progression through phases to reach the outcome of interest. Furthermore, building on previous reports that suggest gender equality is critical to the goals of reduced burden of the disease [31, 32], findings of this study show that gender inequality has an indirect and independent effect on HIV prevalence via human development.

Any change in the parameters included in this model does not necessarily translate to changes in outcome measures, in this case in ART coverage or AIDS related mortality, even if this model was causal. In the next section, we are presenting a summary of the association between the different constructs and the outcome, and presenting the metrics of the associated changes that the model predicts.

Stigma, discrimination, and obstacles to enjoying human rights are hypothesized to be intimately connected to the continuum of care by many direct and indirect links [29, 33]. This study tested those linkages with empirical data, to identify where their effect is most significant, and provided a model to predict the magnitude of their effect. This study corroborates the findings of previous research that negatively associates punitive laws and homophobia with access and use of HIV care [34–36].

Testing service delivery is positively associated with treatment coverage via uptake of HIV testing and counseling. Although not tested in this analysis, the country averages of facilities per population need to account for geographical concentration of people living with HIV.

### Assumptions and limitations

Although this study offers a number of new insights into effects of enablers on the AIDS response, it does have limitations. One of these limitations is the sample size due to the national level of observation and the number of countries having valid observations for each of the variables in the model. The parameter to sample size ratio of this study is 1:4, whereas a 1:5 ratio is recommended [16, 37]. Given the limitation on the sample size, the number of parameters accepted in the model before saturation is reached was also limited. Therefore, a number of parameters were not considered, due to the strength and significance of their effect at the cost of diminishing predictive power of the model. Although limited degrees of freedom could be eased with use of a panel dataset, due to lack availability of data on enablers at multiple points in time, this study relied on a cross-sectional dataset.

The analysis also used some ordinal categorical measurements such as the homophobia index, punitive laws and policies and anti-discrimination conventions. As customary, these ordinal measurements are considered in the analysis as imprecise observations on continuous normally distributed variables. All three ordinal variables considered in this study met the minimum condition of having five categories when taken as continuous variables [38].

Optimal measures to report goodness of fit using Structural Equation Modeling have been intensively debated. As stated by Barret (2007) “(p)roponents of two kinds of approaches to model fit can be identified: those who adhere strictly to the result from a null hypothesis significance test, and those who ignore this and instead index model fit as an approximation function” [39]. The recommendations of this author are to oppose the strict cut-off values for Approximate Fit Indices. Other authors have suggested alternative measures for editorial policies regarding the reports of Structural Equation Modeling [40–42].

For this analysis, the model was re-specified several times and the one judged as “the best goodness of fit” was reported. Alternative models lead to roughly the same interpretations as the current model, but they had lower R^2^ statistics (e.g. R^2^ < 0.4). Removing and adding certain constructs and the associated R^2^ for that model further validated the constructs and model paths. While the R^2^ explains only half of the variance in the outcomes, not all the variables in the model were possible to be included due to the limitation in available empirical data on some constructs. More importantly, since “countries” are the unit of analysis (193 maximum), there was a further limitation in the ratio of observations to parameters (i.e. the more parameters introduced, the more observations needed).

Finally, we decided to keep the marginally significant variables at the 90% confidence interval limit where there was conceptual/theoretical basis to keep them, in particular since the objective of this analysis was exploratory and not causal.

Factor analysis allowed inclusion of a number of related variables in the model as a single latent construct. Governance and logistics were introduced in the model as factors defined by a subset of variables considered. The Human Development Index was used as a single composite, and none of the constructs integrated in the index were analyzed individually.

A number of enablers were identified in the reviewed frameworks for which there was no empirical data available: Community Mobilization, Mass Media, Local Response to Change Risk Environment, Community Centered Design and Delivery, Programme Communication, Management and Incentives, Research and Innovation, Community Systems and Employer Practices, Health Information System, Quality of Care, Safety, and Socio-Cultural Factors.

## Conclusions

This study quantified the direct and indirect contribution of enablers to improved treatment coverage and the associated reduction in AIDS related deaths. While the observational cross-sectional data and statistical model used do not allow inference of causality from enablers to AIDS related mortality, they do provide input into the paths or mechanisms involved in the theoretical models frequently used. Improvements in monitoring of enablers temporally will allow cross-sectional panel data analysis that addresses further ingredients necessary in search of establishing causality. Increasing the number of observations by using data at sub-national units of analysis may allow more parameters to be introduced into the model, even while some of the variables/constructs apply to the whole country. This statistical analysis of cross-sectional data, however, allows establishment of correlations useful for providing metrics on the potential effect of critical enablers on outcomes.

There is a need for further research considering the effects of enablers on HIV incidence. Recognizing the scarcity of national level measures of enablers, we recommend that the availability of data be amplified to improve the analyses aiming at the understanding of these relationships. In addition, costing of programs that strengthen the enabling environment, together with the modeled effect of enablers on outcomes, can support cost-effectiveness studies of programs that consider a multi-sectorial approach to activity planning.

The use of Structural Equation Modeling can be further explored to help in the understanding of the complex direct and indirect contributions of multiple variables and constructs. However, there is need to also explore the use of other models whose goodness of fit can be reported using more traditional measures.

With increasing evidence highlighting gaps in the response, and particular populations being left behind [1, 43], the AIDS response can no longer afford to offer more of the same. Scale-up of efficacious programs are necessary but not sufficient to end AIDS. Clearly where laws impose capital punishment for same-sex activities, HIV testing and treatment cannot be done in isolation of such hostile environments. The same can be stated for stigma and discrimination that may constitute barriers to the access to services (treatment or preventive; personal or population based). This study paves the way for further evaluation of bundling program activities with strengthening of the enabling environment that will make the end of AIDS (as a public health threat) possible.

## Supporting information

S1 Appendix Detailed description of the development and selection of the final model. (PDF)

S1 Dataset Dataset of variables used in the models. Database readable by MPlus for analysis of the structural equation model and the measurement model.
(CSV)

S2 Dataset Dataset of variables used in the models. Dataset readable by MPlus for analysis of the structural equation model and the measurement model as spreadsheet.
(XLSX)

S1 File MPlus diagram file for drawing the measurement model of the two latent constructs: Logistics and Governance. (TXT)

S2 File Mplus input code file for the measurement model of the two latent constructs: Logistics and governance. (TXT)

S3 File MPlus output results file for the measurement model of the two latent constructs: Logistics and governance. (TXT)

S4 File MPlus diagram file for drawing the structural equation model of all constructs and paths. (TXT)

S5 File MPlus input code file for the structural equation model of all constructs and paths. (TXT)

S6 File MPlus output results file for the structural equation model of all constructs and paths. (TXT)
