# Supplementary material for: Direct and indirect effects of enablers on HIV testing, initiation and retention in antiretroviral treatment and AIDS related mortality
Source: PLoS One. 2017 Feb 22;12(2):e0172569. doi: 10.1371/journal.pone.0172569 (PMC5321283; doi:10.1371/journal.pone.0172569)
Supplement: S1 Appendix — (PDF) [file pone.0172569.s001.pdf]

S1 Detailed description of the development and selection of the final model

A systematic search was undertaken to identify frameworks that consider the interaction of enablers and HIV and AIDS program activities and outcomes. The systematic search identified four frameworks that take a broad perspective of the programmatic response and also address the role of enablers in that perspective. The frameworks of interest were the Investment Framework, the Health Systems Framework, the Proximate Determinants Framework, and Program Impact Pathways [1] [2] [3] [4].

The constructs, indicators to measure the constructs, and pathways of interaction elicited in these frameworks were synthesized to formulate a preliminary conceptual model of relationship between enablers, program activities and outcomes (figure 1).

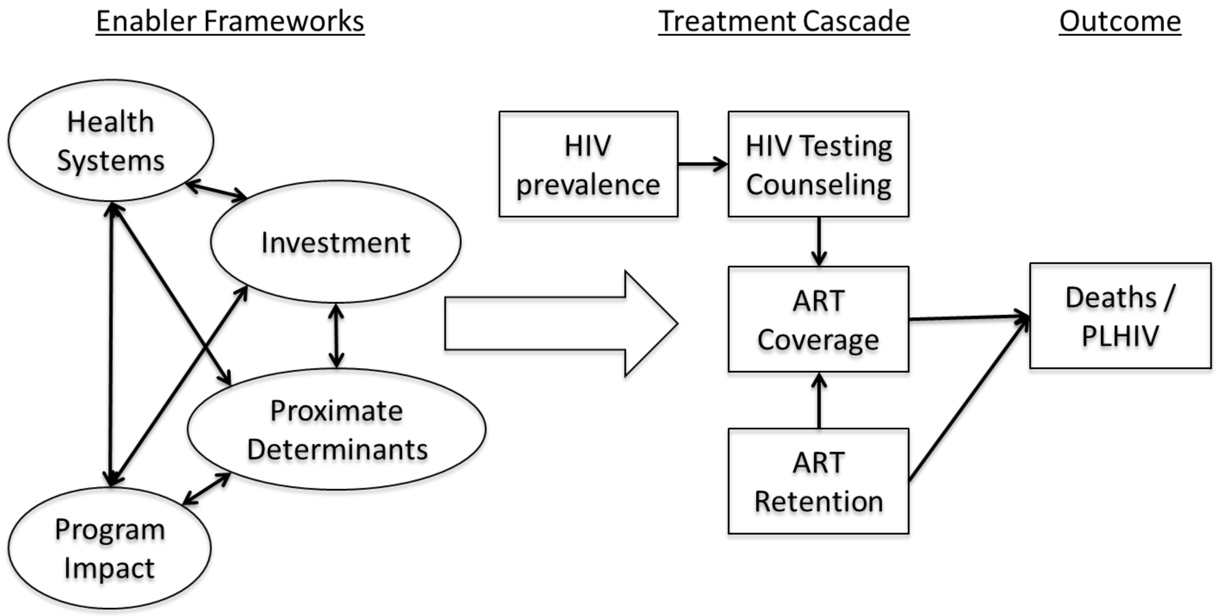

Figure 1 - Conceptual model of relationship between enablers, program activities and outcome

Following in-depth review of the frameworks and associated literature, and interviews with expert informants, databases were identified for measurement of constructs and variables of

conceptual model. The public databases of WHO, UNAIDS, WorldBank, OHCHR, ILGA and UNDP were searched for empirical data that measure the variables of the conceptual model. All data observations were at the national level. Where empirical data was not available to measure the variables of interest, the closest available proxy was selected. If data was unavailable, and an adequate proxy variable could not be identified, the construct was removed from consideration in the conceptual model. Additionally, if data was not comparable across countries, the variable or construct it measured was not considered in the conceptual model. Efforts were made to include the latest available data, allowing for a time delay between the critical enablers and changes in outcomes of treatment coverage and mortality.

A correlational table with bivariate scatterplots was used to check for some pre-conditions of the ensuing analysis, such as conditions of multicollinearity and assumptions of normality, linear relationships and homoscedasticity. Several variables were transformed to improve normality of distribution, where extreme kurtosis and skewness were observed. Specifically, using the box-cox method, HIV Testing and Counseling Facilities per 100k Population ($x^{0.2020202}$), HIV Testing and Counseling per 1000 people ($x^{0.3030303}$), and Out-of-pocket Expenditures on Health Per Capita as a proportion of Gross National Income Per Capita ($x^{0.18181 82}$), were transformed [5]. Estimated number of people living with HIV per total population was transformed with a natural logarithm function.

The correlational table also served to validate the relationship of constructs in the model, including the correlations between HIV Testing and Counseling (HTC) Facilities and Prevalence of HTC ($r=0.72$, $p<0.01$), Prevalence of HTC and ART Coverage ($r=0.53$, $p<0.01$), and ART Coverage and Rate of Mortality ($r=-0.73$, $p<0.01$). The correlational table also helped detect other potential correlations between variables. Where a pairwise relationship between variables was

significant (α level of 0.01), and had conceptual validity, the respective relationship was included in the path model for testing. For example, Logistical Performance was strongly correlated with Effective Governance ($r=0.86$, $p<0.01$) and Human Development ($r=0.74$, $p<0.01$). Human Development was moderately correlated with HIV Prevalence ($r=-0.48$, $p<0.01$). Human Rights Laws and Policies were moderately correlated with the Homophobia Index ($r=0.61$, $p<0.01$) and Homophobia was strongly correlated with Gender Inequality ($r=-0.66$, $p<0.01$). These pathways not previously identified from the conceptual frameworks, but indicated from the correlational strength of variables, were subsequently included in the path model, and tested in the structural equation model.

To identify possible latent constructs in the model, an exploratory factor analysis was performed. The number of factors to extract was determined by a graphical solution, using a Scree test [6]. The Scree test determined four factors to retain for the analysis. The graphical solution to the test shows eigenvalues leveling off at the fourth factor mark (figure 2).

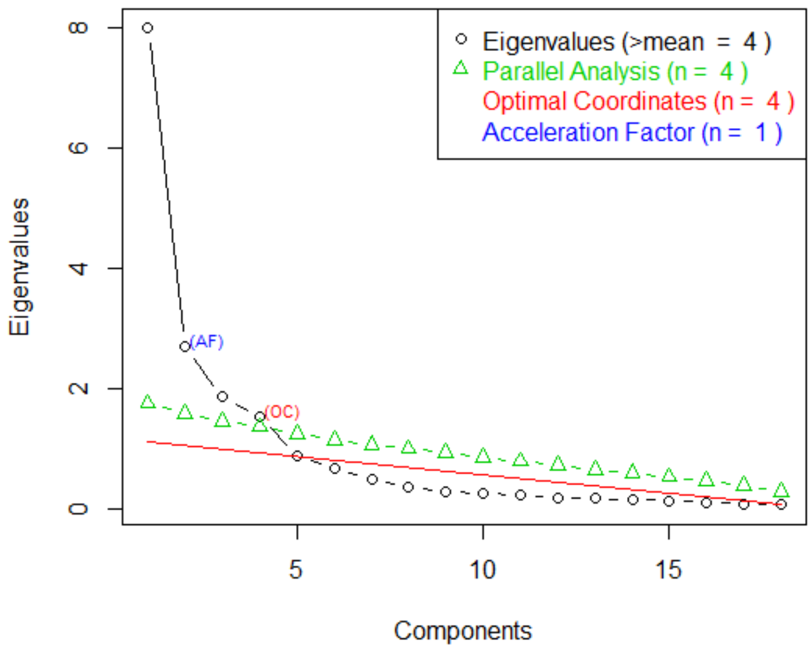

Figure 2 - Scree Test

Factor analysis using the cutoff of four factors confirmed the hypothesis that this number of factors is sufficient (chi-square = 88.45 on 62 degrees of freedom, p-value = 0.0154). Variables with factor loadings of 0.7 or higher were used in the analysis, since this cutoff level corresponds to nearly one half of the variance in the indicator being explained by the factor [7]. Two of the four factors identified by the analysis conceptually fit together. Those two factors were Logistical Enablers (Factor 1) and Governance Enablers (Factor 2) were subsequently considered as latent constructs for the hypothetical model (figure 3).

```
Loadings:
              Factor1 Factor2 Factor3 Factor4
LPI_CUST       0.802   0.351
LPI_INFR       0.819   0.309
LPI_SHIP       0.854
LPI_LGST       0.916
LPI_TRAK       0.848
LPI_TIME       0.784
GOV_RQ_2012    0.363   0.778
GOV_COC_2012           0.850
GOV_EFF_2012   0.452   0.811
GOV_VAA_2012           0.725
GOV_POL_2012           0.661
GOV_ROL_2012   0.311   0.940
lnHIVPREVCALC                  0.820
DPE_1kTHE                      0.876
HR_INDX                                0.731
HOMOINDX                              -0.804

               Factor1 Factor2 Factor3 Factor4
SS loadings      4.879   4.305   1.622   1.426
Proportion Var   0.305   0.269   0.101   0.089
Cumulative Var   0.305   0.574   0.675   0.765

Test of the hypothesis that 4 factors are sufficient.
The chi square statistic is 88.45 on 62 degrees of freedom.
The p-value is 0.0154
```

Figure 3 - Factor analysis

A measurement model was specified for the latent variables of logistics and governance, and estimated by confirmatory factor analysis [8] [9]. Confirmatory factor analysis for the measurement model of the enabler latent constructs indicates an excellent fit of the model with the data. The chi-square test for model fit is non-significant ($\chi 2 = 43{\cdot}719$, df = 34, p-value = 0·1228), the BIC is 97·032, the CFI and TLI are both 0·98, and the RMSEA is 0·07. Figure 1 in the paper provides the standardized parameter estimates for the measurement model. The latent

constructs were introduced into the model where they conceptually fit, and the pathways of the model were subsequently refined to include their effect on program activities, outcomes and other enablers. The hypothetical model containing all observed and latent constructs, and the relationships between the constructs, representing the hypotheses, was evaluated as a structural equation model.

Following the test of consistency of the model with the data, a re-specification determined if the model could be improved. Methods of improving the model included combining measures not previously factored together where there was conceptual validity, dropping measures that showed weak association, and adding correlational paths following transitivity rules or where there was a theoretical rational. The revised model was tested using the steps specified previously. Model modifications were retained if they significantly improved the overall model fit as measured by the test statistic: (1) the overall chi-square test statistics for the null hypothesis that the model is consistent with the data [10], (2) the Bayesian Information Criterion (BIC) for which a negative value indicates the model is preferred over the saturated model allowing for all variables to be intercorrelated [11] [12], (3) the Comparative Fit Index (CFI) and Tucker Lewis Index (TLI) for which values greater than 0.9 are generally taken as indicative of good fit [13] [14], and (4) the Root Mean Square Error of Approximation (RMSEA) for which values less than 0.1 are indicative of good fit in small sample sizes [15] [16]. Once reasonable re-specifications were exhausted, the model was considered final.

References S2

1. Boerma JT, Weir SS. Integrating demographic and epidemiological approaches to research on HIV/AIDS: the proximate-determinants framework. Journal of Infectious Diseases. 2005; 191: p. S61-S67.

2. WHO. World Health Organization. [Online].; 2007 [cited 2013 May 15. Available from: http://www.who.int/healthsystems/strategy/everybodys_business.pdf.

3. Schwartländer B, Stover J, Hallett T, Atun R, Avila C, Gouws E, et al. Towards an improved investment approach for an effective response to HIV/AIDS. The Lancet. 2011 June 11; 377: p. 2031-2041.

4. Weir S, Sabin K, Abdul-Quader A, Au M, Bok L, Butler J, et al. MEASURE Evaluation. [Online].; 2013 [cited 2014 August 19. Available from: http://www.cpc.unc.edu/measure/publications/ms-11-49a.

5. Box GEP, Cox DR. An analysis of transformations. Journal of the Royal Statistical Society. Series B (Methodological). 1964; 26(2): p. 211-252.

6. Cattell RB. The Scree Test For The Number Of Factors. Multivariate Behavioral Research. 1966;: p. 245-276.

7. Lance CE, Butts MM, Michels LC. The Sources of Four Commonly Reported Cutoff Criteria: What Did They Really Say? Organizational Research Methods. 2006;: p. 202-220.

8. Jöreskog KG. A general approach to confirmatory maximum likelihood factor analysis. Psychometrika. 1969 June; 34(2): p. 183-202.

9. Kline RB. Principles and Practice of Structural Equation Modeling: Guilford Press; 2011.

10. Bollen KA. Structural Equations with Latent Variables New York: Wiley; 1989.

11. Raftery A. Bayesian Model Selection in Social Research. Sociological Methodology. 1995; 25: p. 111-163.

12. Schwarz G. Estimating the Dimension of a Model. Annals of Statistics. 1978;: p. 461-464.

13. Bentler PM. Comparative Fit Indexes in Structural Models. Psychological Bulletin. 1990; 107: p. 238-246.

14. Tucker LR, Lewis C. Psychometrika. 1973; 38: p. 1-10.

15. Chen F, Curran PJ, Bollen KA, Kirby J, Paxton P. An Empirical Evaluation of the Use of Fixed Cutoff Points in RMSEA Test Statistic in Structural Equation Models. Sociological Methods & Research. 2008;: p. 462-494.

16. Steiger JH, Lind JC. Statistically-Based Tests for the Number of Common Factors. Annual meeting of the Psychometric Society. 1980; 758.
